# Beyond Surgical Repair in Hypospadias: Genetics, Emerging Technologies, and Lifelong Outcomes—A Narrative Review

**DOI:** 10.3390/children13101330

**Published:** 2026-09-30

**Authors:** Irfan Muhammad Alqadrie, Putu Angga Risky Raharja, Gerhard Reinaldi Situmorang, Irfan Wahyudi, Arry Rodjani, Tariq Abbas

**Affiliations:** 1 Department of Urology, Faculty of Medicine, Universitas Indonesia, Cipto Mangunkusumo Hospital, Jakarta 10430, Indonesia; irfan.muhammad32@ui.ac.id (I.M.A.); putu.angga31@ui.ac.id (P.A.R.R.); gerhard.reinaldi01@ui.ac.id (G.R.S.); irfan.wahyudi@ui.ac.id (I.W.); arry.rodjani@ui.ac.id (A.R.); 2 Pediatric Urology Section, Sidra Medicine, Doha P.O. Box 26999, Qatar; 3 College of Medicine, Qatar University, Doha P.O. Box 2713, Qatar; 4 Weill Cornell Medicine-Qatar, Doha P.O. Box 24144, Qatar

**Keywords:** hypospadias, multidisciplinary care, pediatric urology, genetics, disorders of sex development, artificial intelligence, digital health, psychosocial support, precision medicine

## Abstract

**Highlights:**

**What are the main findings?**

- The review identifies objective phenotyping as a useful complement to anatomical classification and supports phenotype-driven endocrine/genetic assessment, which should be considered in proximal hypospadias (including boys with descended testes) and is most strongly indicated when there are features suggestive of a disorder of sex development (DSD), familial disease, or other associated abnormalities.
- Emerging artificial intelligence (AI), quantitative tissue assessment, registries, and biobanks may improve standardisation and research integration, but their clinical use remains investigational and requires prospective validation.

**What are the implications of the main findings?**

- A phenotype-driven approach is preferable to universal multidisciplinary or genetic evaluation: straightforward distal hypospadias can generally remain within routine pediatric urologic care, whereas proximal hypospadias warrants consideration of endocrine/genetic evaluation, with formal DSD-team assessment when atypical or associated features are present.
- Long-term outcome assessment should extend beyond childhood, particularly for complex or previously complicated cases, while emerging digital and psychosocial tools should be used as adjuncts to—not substitutes for—clinical expertise.

**Abstract:**

**Background/Objectives:** Hypospadias is a heterogeneous congenital anomaly. This selected narrative review examines emerging domains that may complement conventional surgical care, with particular emphasis on complex, proximal, atypical, familial, or disorder of sex development (DSD)-associated presentations and on long-term outcomes. **Methods:** A structured narrative search of PubMed/MEDLINE, Scopus, and Web of Science was conducted for English-language human or clinically applicable translational literature published from January 2000 through December 2025, supplemented by reference screening and targeted updating of genetics, non-operative management, and adult-outcome literature. **Results:** Objective phenotyping may improve anatomical description and research comparability. Endocrine/genetic evaluation should be considered in proximal hypospadias, including boys with descended testes, and is most clearly indicated when accompanied by cryptorchidism, micropenis, undervirilisation, familial disease, or other features suggestive of DSD; isolated distal hypospadias without such features does not generally require genetic or formal multidisciplinary DSD assessment. Limited evidence also supports explicit consideration of non-operative management in selected mild distal cases, while studies of uncorrected distal hypospadias report heterogeneous functional and cosmetic outcomes. Artificial intelligence (AI), quantitative tissue assessment, registries, and biobanks remain investigational. Long-term studies highlight sexual, reproductive, psychosocial, and late surgical outcomes that may extend into adulthood. **Conclusions:** The literature supports a selective, phenotype-driven framework rather than a universal multidisciplinary model, with particular attention to complex/atypical disease and lifelong outcome assessment.

## 1. Introduction

Hypospadias is one of the most common congenital anomalies affecting male genital development, with reported prevalence varying across populations [1,2]. It is characterised by an ectopic urethral opening on the ventral aspect of the penis and may be associated with ventral curvature, abnormal foreskin development, and urethral plate abnormalities [2,3]. Most cases are distal, whereas proximal forms represent a smaller but clinically more complex subgroup [2]. Surgical reconstruction remains an important treatment for selected patients [4], but management decisions increasingly consider functional symptoms, curvature, appearance, patient/family preferences, and the potential consequences of intervention as well as non-operative observation in selected mild cases.

Historically, hypospadias severity and treatment planning have been primarily based on anatomical parameters, particularly meatal location. Although anatomical classification remains essential for surgical decision-making, it does not fully capture the complexity of the condition or reliably predict surgical difficulty and long-term outcomes. Recent evidence suggests that additional parameters reflecting urethral development, tissue characteristics, and associated anomalies may provide a more comprehensive assessment. Objective classification systems, such as the urethral defect ratio (UDR), have been proposed to complement traditional anatomical descriptions by quantifying developmental deficiency and demonstrating associations with curvature severity, urethral plate characteristics, and overall disease complexity [5]. Furthermore, long-term outcomes extend beyond surgical success and include urinary function, sexual health, fertility considerations, body image, psychological well-being, and quality of life [6]. These factors emphasise the need for a more holistic approach that incorporates both clinical outcomes and patient-reported experiences.

The evolving understanding of hypospadias has highlighted several emerging domains that may contribute to more individualised and coordinated care. Genetic research indicates that a subset of patients, particularly those with proximal hypospadias, associated anomalies, or familial disease, may have underlying molecular abnormalities involving pathways related to sexual differentiation and urethral development [7,8,9,10,11]. Advances in genetic testing, including chromosomal microarray analysis and next-generation sequencing, provide opportunities for improved diagnostic stratification and counselling in selected patients [8,10]. Similarly, technological innovations are transforming hypospadias assessment: artificial intelligence (AI)-based image analysis has shown potential for standardised classification, while multidisciplinary phenotyping and quantitative tissue assessment may add biological context to anatomical evaluation [12,13,14]. In parallel, family-centred approaches, including structured education and shared decision-making tools, have gained importance as families navigate complex treatment decisions and expectations [15,16,17]. Psychosocial support is also relevant because hypospadias and its treatment may affect body image, emotional well-being, and quality of life across childhood, adolescence, and adulthood [18,19,20].

The literature therefore supports a multidimensional but selective model of care. Objective anatomical phenotyping, targeted biological evaluation, digital innovation, proportionate family education, psychosocial assessment, and long-term outcome surveillance can complement surgical expertise. Importantly, these domains should not be interpreted as a requirement for every child with hypospadias. In particular, endocrine/genetic evaluation should be considered in proximal hypospadias, whereas formal multidisciplinary review for a disorder of sex development (DSD) is most relevant when the phenotype raises concern for an underlying DSD or another associated condition [8,9,10,11].

This narrative review provides a selected interpretation of emerging evidence rather than a comprehensive systematic overview of all hypospadias literature. It focuses on objective phenotyping, selective genetic/DSD evaluation, prenatal diagnosis, artificial intelligence, digital pathology, family-centred decision-making, psychosocial and adult outcomes, non-operative management in selected distal disease, and research infrastructure, while identifying areas in which evidence remains heterogeneous or incomplete.

## 2. Materials and Methods

### 2.1. Literature Search Strategy

A structured narrative review approach was adopted to identify and synthesise contemporary evidence relevant to selected emerging domains in hypospadias care. Searches were performed in PubMed/MEDLINE, Scopus, and Web of Science for publications from January 2000 through December 2025. The review was intentionally selective and was not designed as a systematic review or meta-analysis.

The search strategy combined controlled vocabulary and free-text terms related to hypospadias and the predefined domains. Concepts included: (“hypospadias” OR “hypospadiology”) AND (“phenotyping” OR “severity” OR “genetics” OR “genomic” OR “whole exome” OR “GWAS” OR “SNP” OR “disorders of sex development” OR “prenatal diagnosis” OR “artificial intelligence” OR “digital health” OR “digital pathology” OR “psychosocial outcomes” OR “quality of life” OR “fertility” OR “paternity” OR “adult outcomes” OR “untreated” OR “non-operative” OR “shared decision-making”).

Additional relevant publications were identified through manual screening of reference lists and targeted citation searching. Particular attention was given during revision to genetic studies using GWAS and whole-exome sequencing, the literature addressing mild/untreated distal hypospadias, long-term reoperation data, sexual and reproductive outcomes, and adult management of late complications. The review was updated to December 2025 so that these domains were represented in the final synthesis.

### 2.2. Study Selection

Eligible publications included original research studies, narrative and systematic reviews, consensus statements, clinical guidance documents, and relevant book chapters addressing contemporary hypospadias evaluation, management, or long-term outcomes. Studies were included when they contributed directly to one or more predefined domains. Publications were limited to English-language human studies or clinically applicable translational research. For the biological/DSD domain, particular emphasis was placed on proximal or atypical hypospadias and on hypospadias accompanied by cryptorchidism, micropenis, undervirilisation, genital ambiguity, endocrine abnormalities, or familial clustering. Isolated distal hypospadias without such features was not treated as an indication for genetic testing or formal multidisciplinary DSD assessment. Studies unrelated to hypospadias, basic laboratory work without clinically relevant implications, and technical surgical descriptions without relevance to the selected review domains were excluded. Findings were synthesised narratively rather than quantitatively.

### 2.3. Scope of Review

This narrative review evaluates selected interconnected domains rather than attempting to cover the entire hypospadias literature. The scope was deliberately narrowed toward emerging assessment, biological stratification, technology, family-centred care, psychosocial and reproductive outcomes, non-operative management in selected distal disease, adult complications, and research infrastructure (Table 1). Accordingly, statements are intended as an expert synthesis of selected evidence and should not be interpreted as universal management recommendations.

### 2.4. Review Process Overview

**Figure 1:**
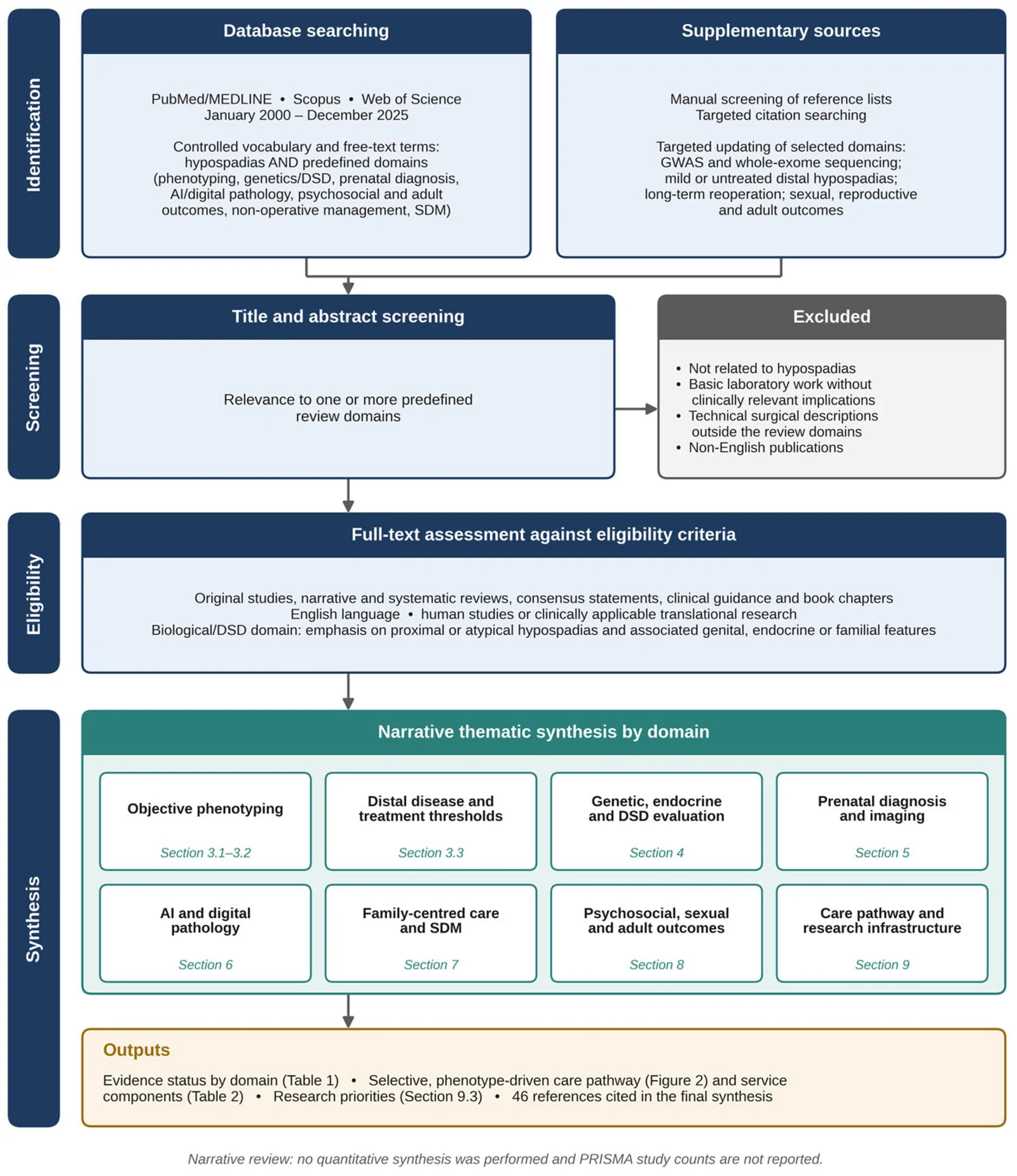
Overview of the narrative review process. Publications were identified through database searching and supplementary sources, screened for relevance to the predefined domains, assessed in full text against the eligibility criteria, and synthesised narratively by domain. Because this was a narrative rather than a systematic review, no quantitative synthesis was performed, and PRISMA study counts are not reported. AI, artificial intelligence; DSD, disorder of sex development; GWAS, genome-wide association study; SDM, shared decision-making.

The identification, screening, eligibility assessment, and synthesis steps of the review are summarised in Figure 1.

## 3. Evolution of Hypospadias Assessment: From Anatomy-Based Classification to Multidimensional Phenotyping

### 3.1. Traditional Anatomical Assessment

The assessment of hypospadias has traditionally relied on anatomical characteristics, with meatal position representing the most commonly used descriptor. Hypospadias is generally classified as distal, midshaft, or proximal according to meatal position [2,3]. Most cases are distal and often have a relatively limited associated anatomical burden, whereas proximal disease is less common and more frequently associated with marked curvature, tissue deficiency, cryptorchidism, or other atypical findings [2,3].

However, hypospadias is not solely defined by meatal position. The condition represents a spectrum of developmental abnormalities involving the urethra, glans, penile tissues, and ventral structures. The presence and degree of ventral curvature (chordee), urethral plate characteristics, glans configuration, and penile tissue development may substantially influence surgical complexity and outcomes [3]. In particular, curvature assessment is an essential component of preoperative evaluation because significant chordee may require additional reconstructive procedures beyond urethral tubularisation.

The urethral plate has also gained increasing recognition as an important determinant of surgical decision-making. Its width, quality, and degree of development may influence the feasibility of plate-preserving techniques and the choice between single-stage and staged reconstruction. Although experienced surgeons incorporate these factors during operative planning, traditional classifications based primarily on meatal position may not fully capture the underlying anatomical variability among patients with similar meatal locations.

The limitations of anatomy-based classification have become increasingly apparent as the field has moved toward more individualised treatment strategies. Patients categorised within the same anatomical group may demonstrate substantial differences in curvature severity, tissue characteristics, associated anomalies, and postoperative outcomes. Therefore, contemporary assessment approaches aim to complement traditional descriptions with objective and multidimensional phenotyping systems that better represent the complexity of hypospadias.

### 3.2. Objective Phenotyping Systems

To address the limitations of traditional anatomical classification, several objective assessment systems have been developed to provide more comprehensive characterisation of hypospadias severity. These approaches aim to integrate multiple anatomical parameters rather than relying exclusively on meatal position, facilitating improved communication, research standardisation, and potentially more individualised surgical planning.

The urethral defect ratio (UDR) represents an embryology-informed approach that quantifies the extent of urethral developmental deficiency by measuring the distance between the glanular knobs and the bifurcation of the corpus spongiosum relative to stretched penile length [5]. This objective parameter has demonstrated associations with important anatomical features, including curvature severity and urethral plate characteristics, and provides a reproducible method for describing developmental deficiency beyond meatal location alone. Importantly, UDR is intended to complement existing clinical assessments rather than replace established classification methods.

The Glans–Urethral Meatus–Shaft (GMS) score incorporates three anatomical components—glans configuration, meatal position, and penile shaft characteristics—to provide a structured evaluation of hypospadias severity. By combining multiple relevant features, GMS offers a more comprehensive assessment of the anatomical phenotype and has been used in studies evaluating outcomes and surgical complexity [21].

The Hypospadias Objective Penile Evaluation (HOPE) score was developed to provide a standardised assessment of penile appearance based on objective anatomical criteria. This tool aims to improve consistency in evaluating cosmetic outcomes and facilitate comparison between clinical studies by reducing variability associated with subjective assessment [22].

The Plate Objective Scoring Tool (POST) further contributes to objective characterisation by quantifying urethral plate quality using reproducible anatomical landmarks [23]. Such tools highlight the increasing importance of standardised phenotyping in contemporary hypospadias research and clinical practice.

Collectively, these systems reflect a shift from a purely location-based classification toward a multidimensional understanding of hypospadias. Rather than replacing traditional anatomical descriptions, objective phenotyping tools provide complementary information that may improve severity assessment, facilitate research collaboration, and support more individualised approaches to counselling and management. Future integration of anatomical scoring with genetic, biological, and patient-reported data may further advance precision-based care in hypospadias.

### 3.3. Distal Hypospadias and Evolving Treatment Thresholds

The majority of hypospadias cases are distal, and this subgroup should not be conflated with the selected complex presentations that form the principal focus of this review. Recent literature has renewed discussion about whether every mild distal case requires surgical correction, particularly when urinary and sexual function are expected to be preserved. A 2025 scoping review concluded that available evidence supports consideration of non-operative management in selected mild cases, while noting the paucity of research and the need to balance parental wishes with clinical guidance [24]. Conversely, a social media-based survey found that men with untreated hypospadias reported worse sexual and urinary outcomes and lower satisfaction with penile appearance than controls, although adverse outcomes were less frequent in milder forms [25], and a series of 51 men with uncorrected distal hypospadias presenting to a specialty centre reported frequent urine spraying, penile curvature, painful sexual activity, and concern about appearance [26]. Both designs are susceptible to selection bias, but together these studies illustrate that the natural history of untreated distal disease is heterogeneous. The appropriate threshold for intervention therefore remains individualised and should consider anatomy, curvature, urinary function, appearance, family/patient preferences, and the quality of available long-term evidence rather than a universal rule.

## 4. Biological Stratification in Proximal/Atypical Hypospadias and Hypospadias with Features Suggestive of DSD

Hypospadias has a multifactorial developmental basis involving genetic susceptibility, androgen signalling, environmental influences, and local tissue development [7,8,9]. The majority of isolated cases—particularly distal forms—do not have an identifiable monogenic disorder. Biological evaluation becomes more clinically relevant when hypospadias is proximal or atypical, familial, or accompanied by cryptorchidism, micropenis, genital ambiguity, undervirilisation, endocrine abnormalities, or other findings suggestive of DSD [8,9,10,11].

### 4.1. Genetic Contributions to Hypospadias

Normal male urethral development requires coordinated regulation of androgen signalling, sex-determination pathways, and tissue differentiation processes. Genetic alterations affecting these pathways have been implicated in hypospadias, particularly in patients with severe phenotypes or associated abnormalities [7,8,9].

The androgen receptor (AR) gene is clinically relevant because androgen signalling plays a central role in masculinisation and urethral development. Variants affecting androgen receptor function may impair responsiveness to circulating androgens and contribute to genital developmental abnormalities, including hypospadias, particularly when associated with other features of undervirilisation [7,8,9].

The SRY (sex-determining region Y) gene is a key regulator of testicular differentiation. Alterations involving SRY and related sex-determination pathways may occur in disorders of sex development (DSD), in which hypospadias can represent one component of a broader developmental phenotype requiring multidisciplinary evaluation [8,10].

Beyond candidate genes, genomic approaches have broadened the field. Genome-wide association studies (GWAS) have identified susceptibility loci in developmental pathways, while whole-exome sequencing has identified potentially relevant rare variants and mutational hotspots, particularly in severe disease [27,28]. These studies also reinforce the polygenic and heterogeneous nature of hypospadias and the importance of integrating genomic findings with phenotype, embryology, and environmental context rather than assuming a single causal gene.

Familial studies have further expanded understanding of the genetic architecture of hypospadias. Recent whole-genome sequencing of seven multiplex families has identified rare variants in genes potentially involved in urethral development and developmental pathways while also demonstrating substantial genetic heterogeneity [11]. These findings support integration of genomic, phenotypic, and environmental data rather than a single-candidate-gene model.

### 4.2. Clinical Role of Genetic Testing

The clinical application of genetic evaluation requires appropriate patient selection. Genetic testing is not indicated for every child with isolated distal hypospadias, and proximal meatal position alone should not automatically be equated with DSD. Nevertheless, proximal hypospadias is itself a marker of increased genetic risk. In one series, identifiable genetic differences were found in 10% of boys with proximal hypospadias and bilaterally descended testes, compared with 33% of those with cryptorchidism [10]; in another cohort of boys who underwent endocrine/genetic evaluation, genetic anomalies were identified in 17% of those with bilaterally descended testes and 53% of those with at least one undescended testis [29]. These data support discussing endocrine/genetic evaluation with the families of all boys with proximal hypospadias, with testing most strongly indicated when proximal/atypical hypospadias is accompanied by additional findings that raise concern for disordered sex development or another genetic condition.

Patients who may particularly benefit from endocrine/genetic evaluation include those with:

- Proximal hypospadias, especially when associated with additional genital abnormalities;
- Cryptorchidism, particularly with one or more non-palpable testes [29], which may indicate abnormalities of sexual differentiation;
- Micropenis or evidence of undervirilisation;
- Features suggestive of disorders of sex development (DSD), including atypical genital development or hormonal abnormalities;
- Familial hypospadias, particularly when multiple affected relatives are identified.

Modern evaluation may include chromosome-level testing, targeted panels, whole-exome sequencing, or other sequencing strategies selected according to phenotype and local diagnostic pathways [8,10,27,28]. Genome-wide studies and sequencing have expanded knowledge of susceptibility loci and rare variants, but diagnostic yield remains variable, and findings such as variants of uncertain significance require careful interpretation. Genetic testing should therefore be embedded within an appropriate clinical pathway rather than used as a universal screening test.

The primary clinical value of genetic evaluation is to clarify selected atypical phenotypes, identify associated conditions, support counselling, and determine whether further endocrine or DSD evaluation is warranted. When a DSD is excluded, and no other indication for multidisciplinary review remains, ongoing care does not require a formal multidisciplinary council solely because the child has proximal hypospadias. Referral should remain phenotype- and problem-driven.

### 4.3. Future Directions: Toward Genotype–Phenotype Integration and Precision Medicine

Future advances in hypospadias care will likely depend on integrating genetic information with detailed clinical phenotyping and longitudinal outcomes. Dedicated biobanks that combine genomic data with standardised anatomical assessments, surgical details, tissue characteristics, and patient-reported outcomes represent one approach to studying disease heterogeneity and developing future prognostic models [11,30].

Genotype–phenotype correlation studies may help identify relationships between specific genetic alterations, anatomical severity, surgical complexity, and long-term outcomes. Such approaches may allow improved patient stratification and more individualised counselling regarding expected clinical trajectories.

Large collaborative databases and multidisciplinary research platforms may further accelerate discovery by linking genetic, biological, and clinical information. Integration of genomic data with emerging technologies such as artificial intelligence and predictive analytics may ultimately support precision medicine approaches in hypospadias, although prospective validation and ethical considerations remain essential before widespread clinical implementation.

Overall, biological evaluation should be selective and phenotype-driven. Proximal hypospadias warrants consideration of endocrine/genetic evaluation, and proximal or atypical presentations with associated genital or endocrine findings warrant DSD-oriented assessment, whereas isolated distal hypospadias generally remains within routine pediatric urologic care. Future genotype–phenotype studies may improve the precision of this selection process.

## 5. Prenatal Diagnosis and Early Multidisciplinary Counselling

Prenatal recognition of hypospadias is primarily relevant when imaging suggests severe genital anomaly, atypical genital development, or associated abnormalities. Its main clinical value is counselling and appropriate postnatal referral rather than determining surgical management before birth.

### 5.1. Current Imaging Approaches

Ultrasonography remains the primary imaging modality for prenatal assessment of fetal genital anatomy because of its safety, accessibility, and widespread availability. Sonographic findings that may raise suspicion for hypospadias include abnormal penile orientation, shortened penile appearance, ventral curvature, atypical external genital configuration, and, in severe cases, the characteristic ‘tulip sign’ [31,32,33].

The diagnostic performance of prenatal ultrasound varies with gestational age, fetal position, operator experience, severity of hypospadias, and image quality [31,32]. Distal and less severe forms are particularly challenging to identify prenatally because subtle abnormalities of the urethral opening may not be visualised reliably. Fetal genital assessment may also be limited by unfavourable fetal position, reduced amniotic fluid volume, and limitations in anatomical resolution.

Therefore, prenatal ultrasound should be considered a valuable screening and diagnostic support tool rather than a definitive assessment of postnatal severity. Confirmation of hypospadias characteristics and detailed anatomical evaluation remain dependent on postnatal examination by experienced clinicians.

### 5.2. Role of Advanced Imaging

Advanced imaging modalities, particularly fetal magnetic resonance imaging (MRI), may provide complementary anatomical information in selected complex fetal genital anomalies. MRI offers additional soft-tissue characterisation and may be useful when genital ambiguity or associated anomalies raise concern for a disorder of sex development [34].

Fetal MRI is not a routine investigation for isolated suspected hypospadias. Its role is best considered selectively when ultrasound is inconclusive or when complex genital or associated anomalies require clarification, particularly when a DSD or another fetal anomaly is suspected [34].

The future role of advanced imaging may involve integration with other clinical information, including genetic findings and postnatal phenotyping, to support more comprehensive characterisation of complex cases. However, further evidence is required before advanced imaging can be incorporated into standardised prenatal pathways.

### 5.3. Clinical Value of Early Identification

The primary value of prenatal recognition of hypospadias lies in improving communication, counselling, and coordination of care rather than directly altering surgical outcomes. Early identification provides an opportunity to prepare families by explaining the condition, discussing potential postnatal evaluation, and addressing uncertainty regarding severity and treatment options.

In cases where hypospadias is associated with other findings suggestive of disorders of sex development, early recognition may facilitate timely referral to multidisciplinary teams involving pediatric urologists, endocrinologists, geneticists, and specialised imaging services. This coordinated approach may reduce delays in diagnosis, support appropriate investigations, and provide families with consistent information during an emotionally challenging period.

Furthermore, prenatal counselling allows healthcare providers to establish realistic expectations by emphasising that definitive assessment of anatomy, surgical planning, and long-term outcomes requires postnatal evaluation. Early involvement of specialist teams can therefore serve as a foundation for patient-centred care pathways extending from prenatal assessment through childhood and adolescence.

Overall, prenatal imaging represents an important component of an integrated hypospadias care pathway. Its greatest contribution is not the prediction of surgical success but the facilitation of informed counselling, coordinated referral, and early multidisciplinary engagement for affected families.

## 6. Digital Transformation of Hypospadias Care

Technological advances are increasingly influencing the assessment and management of hypospadias by providing opportunities for improved standardisation, objective evaluation, and data-driven decision support. Artificial intelligence (AI), digital health platforms, and computational pathology represent emerging tools that may complement clinical expertise by improving assessment consistency and enabling future precision-based approaches. However, these technologies remain evolving fields, and their integration into routine clinical practice requires robust validation, appropriate regulation, and continued collaboration between clinicians, engineers, and researchers.

### 6.1. Artificial Intelligence in Assessment and Decision Support

Clinical assessment of hypospadias relies heavily on expert interpretation of anatomical features, which may vary with experience and individual judgement. Early studies have explored machine-learning and image-recognition approaches for identifying hypospadias features and assisting with severity classification, demonstrating the potential of AI to support more objective assessment [12,13].

One area of investigation is automated evaluation of anatomical parameters relevant to structured hypospadias assessment. AI-assisted image analysis may improve reproducibility and reduce interobserver variability, but these applications remain dependent on representative training data, transparent measurement definitions, and external validation [12,13].

Mobile-based AI applications represent another emerging area of interest. Standardised clinical photographs analysed using validated algorithms could potentially support assessment, documentation, education, and referral workflows. However, implementation requires careful consideration of image quality, privacy, consent, algorithmic bias, and external validation across diverse populations [13].

The AI-PEDURO collaborative illustrates a broader effort to synthesise and maintain evidence on artificial intelligence in pediatric urology through a living scoping review and online repository; its initial report included 59 studies and found that clinical translation of these models remains limited [35]. Such initiatives may help define research priorities and validation standards as AI applications develop.

Despite these promising developments, AI should currently be considered an adjunctive decision-support tool rather than a replacement for expert clinical assessment. Human interpretation remains essential because surgical planning requires integration of multiple factors, including anatomy, tissue quality, patient characteristics, family preferences, and surgeon expertise. Future studies should focus on prospective validation, transparency of algorithms, clinical utility, and equitable implementation before widespread adoption.

### 6.2. Digital Histopathology and Tissue Assessment

The biological characteristics of hypospadias tissues, particularly the urethral plate and surrounding structures, may influence surgical decision-making and postoperative outcomes. Histological studies have characterised urethral plate composition, and more recent multidisciplinary phenotyping work has incorporated quantitative image and tissue analysis into broader biological assessment [14,36].

Computational analysis of digitised histological or tissue images may allow more quantitative assessment of features such as fibrosis, inflammation, and tissue organisation. In hypospadias, however, this remains primarily a research approach rather than an established clinical prognostic tool [14].

Quantitative tissue analysis may ultimately help link biological features with anatomy and postoperative outcomes, but current evidence is insufficient to define validated tissue biomarkers for routine surgical decision-making [14].

Integration of tissue data with anatomical phenotyping, genetic information, operative details, and longitudinal outcomes represents an emerging direction toward comprehensive biological profiling. Prospective, standardised datasets will be required before such models can be used for individual risk prediction [14,30].

Accordingly, digital tissue analysis should currently be regarded as an investigational component of hypospadias research. Its potential value lies in generating reproducible biological measurements that may complement, rather than replace, clinical and anatomical assessment [14].

Together, artificial intelligence and digital pathology represent important components of the evolving digital transformation of hypospadias care. Their greatest potential lies in enhancing objective assessment, improving communication, and supporting precision-based research while maintaining expert clinical judgement as the foundation of patient management.

## 7. Family-Centred Care and Shared Decision-Making

Family-centred communication is relevant across hypospadias care, but the intensity of support should be proportionate to the clinical situation. Families of children with straightforward distal hypospadias may require clear routine counselling and informed consent, whereas complex, atypical, recurrent, or preference-sensitive cases may benefit from structured decision aids, additional education, or psychosocial support.

### 7.1. Parental Information Needs

The diagnosis of hypospadias can generate anxiety and uncertainty among parents because it involves genital development, future function and appearance, and decisions about surgery. Qualitative work has documented substantial parental information needs during this process [15].

Treatment decisions are most complex when anatomy, curvature, urethral plate characteristics, potential complications, or uncertainty about long-term outcomes create more than one reasonable pathway. In straightforward distal cases, counselling may be comparatively focused; in complex or atypical cases, families may need more detailed discussion of staged procedures, complications, additional interventions, and long-term follow-up [15].

Studies of parental experiences and postoperative decision regret indicate that uncertainty, information needs, and treatment outcomes may contribute to decisional conflict or regret [15,16,17]. Effective counselling should therefore provide balanced information regarding the condition, treatment options, expected benefits, potential risks, and the possibility that outcomes may evolve over time.

Clear communication should therefore be calibrated to the individual case. For complex disease, discussions should include functional outcomes, cosmetic considerations, possible reoperations, sexual and reproductive outcomes, and long-term follow-up. For uncomplicated distal disease, routine informed counselling may be sufficient without implying a need for formal multidisciplinary support.

### 7.2. Digital Education Tools and Decision Support Resources

Digital education platforms have emerged as valuable tools to complement direct clinician–family communication by providing accessible, standardised, and easily revisited information. Families often seek additional information outside clinical consultations, and structured digital resources may help address knowledge gaps and improve understanding of hypospadias.

The Hypospadias Hub is an example of a web-based, parent-centred decision-support resource developed to improve knowledge and support shared decision-making in hypospadias care [37,38]. Such platforms may help parents understand clinical terminology, prepare questions for consultations, and participate more actively in discussions with healthcare providers.

Decision aids can support families facing complex treatment choices by presenting options, expected outcomes, uncertainties, and potential complications in a structured manner. Early user-centred and multisite evaluations of hypospadias decision-support tools have demonstrated feasibility and acceptability, while larger studies are still needed to determine their effects on longer-term decisional and clinical outcomes [37,38].

However, digital resources should be considered complementary rather than substitutes for individualised counselling. The interpretation of information requires clinical context, particularly because hypospadias management involves individualised decisions based on anatomy, patient factors, and surgical expertise. Future development of digital resources should focus on accessibility, cultural adaptation, multilingual content, and evaluation of their impact on knowledge, anxiety, and decisional confidence.

### 7.3. Shared Decision-Making and Expectation Alignment

Shared decision-making is an important component of pediatric surgical care when more than one reasonable management pathway exists or when outcomes involve both objective and preference-sensitive considerations. In hypospadias, it involves collaboration between clinicians and families while respecting family values, preferences, and expectations [15,37].

Effective shared decision-making requires informed consent that extends beyond procedural explanation. Families should receive understandable information regarding the nature of the condition, available treatment approaches, potential complications, likelihood of additional procedures, and the importance of long-term follow-up. This approach allows parents to make decisions based on realistic expectations rather than fear or incomplete information.

Expectation alignment is particularly important in hypospadias because perceptions of success may differ between surgeons, parents, and eventually patients themselves. While surgeons may focus on anatomical and functional outcomes, families may place additional importance on appearance, psychological well-being, and future quality of life. Recognising these different perspectives supports more patient-centred care.

Ultimately, family-centred counselling, supported by educational resources and shared decision-making principles, represents an essential component of integrated hypospadias management. By improving understanding, reducing uncertainty, and aligning expectations, these approaches strengthen the partnership between families and healthcare teams throughout the treatment pathway.

## 8. Psychosocial Health and Lifelong Outcomes

Hypospadias management has traditionally focused on anatomical correction during early childhood; however, increasing recognition of patient-reported outcomes highlights the importance of addressing psychosocial well-being throughout the lifespan. The impact of hypospadias extends beyond surgical outcomes and may involve body image, self-perception, emotional health, relationships, and concerns related to future sexual and reproductive function. A comprehensive approach therefore requires longitudinal assessment and support extending from childhood through adolescence and adulthood.

### 8.1. Childhood and Adolescence: Body Image, Stigma, and Psychological Well-Being

Children with hypospadias may encounter psychosocial concerns as they develop greater awareness of their bodies and differences from peers. Although many individuals adapt well following repair, some report concerns related to genital appearance, disclosure, or future function [18,19]. These issues may become more salient during school years and adolescence, when body image, peer relationships, and social acceptance assume greater importance.

Body image represents an important component of psychosocial health in patients with hypospadias. Perceptions of genital appearance may be influenced by surgical outcomes, the severity of the original condition, postoperative complications, family communication, social context, and individual psychological factors [18,19]. These influences underscore that psychosocial adjustment is not determined by anatomical outcomes alone.

Anxiety may also arise from uncertainty regarding future urinary, sexual, or reproductive outcomes. Early identification of psychological concerns through developmentally appropriate assessment allows timely intervention and may support healthier coping strategies. Integration of psychological professionals within multidisciplinary teams can facilitate counselling for patients and families, particularly during important developmental transitions.

### 8.2. Sexual Function and Transition to Adult Care

As patients progress into adolescence and adulthood, concerns related to sexual function, intimate relationships, genital self-perception, fertility, paternity, and reproductive health may become increasingly relevant. Long-term studies indicate that many men report satisfactory outcomes; however, in one adolescent and young adult cohort, a disturbed urinary and/or suboptimal sexual functional outcome was observed in 52.9% of patients, and proximal hypospadias was associated with impaired sexual function [39,40,41].

Adolescence represents a critical period when patients begin to develop greater independence and may seek information about their condition, previous surgery, genital appearance, and future sexual health. However, many patients do not receive structured follow-up during this transition period, resulting in gaps in counselling and support.

Transition clinics or structured pathways linking pediatric and adult healthcare providers may provide an opportunity to address evolving needs, including urinary function, sexual function, body image, psychological well-being, and reproductive concerns. Such models promote continuity of care and recognise hypospadias as a condition requiring lifelong consideration rather than a childhood surgical event alone.

Adult follow-up is particularly relevant for patients with complex hypospadias, previous complications, multiple surgeries, persistent curvature or urinary symptoms, or concerns regarding sexual or reproductive function. Adult reconstructive urologists may encounter strictures, fistulae, diverticula, recurrent curvature, and other late complications, sometimes years after childhood treatment [42].

### 8.3. Adult Presentation and Management of Late Complications

Late complications may present for the first time in adolescence or adulthood, even when childhood follow-up was considered satisfactory. Long-term series have documented secondary surgery after both distal and proximal repair, with longer follow-up revealing more events than early postoperative assessment alone [40,43]. Adult reconstructive management may involve urethral stricture disease, fistula, diverticulum, recurrent curvature, sexual dysfunction, or concerns about genital appearance. Contemporary reviews emphasise that adult patients may require technically demanding reconstruction because of tissue deficiency and prior operations [42]. A lifelong model should therefore provide a mechanism for transition to adult urology and access to reconstructive expertise when symptoms or patient priorities emerge.

Fertility and paternity deserve explicit consideration. Studies of young adult men born with hypospadias have examined endocrine and seminal parameters, while adolescent/young adult cohorts have highlighted sexual function and satisfaction as important long-term outcomes [39,40,41]. A recent systematic review and meta-analysis likewise supports continued attention to adult sexual function and standardised long-term outcome assessment [44]. These data do not imply poor outcomes for all patients; rather, they support individualised counselling and transition care, particularly for severe or previously complicated disease.

### 8.4. Patient-Reported Outcomes and Quality of Life Assessment

Traditional evaluation of hypospadias outcomes has relied heavily on surgeon-reported measures, including complication rates, anatomical appearance, and technical success. While these outcomes remain important, they do not fully capture the patient’s experience or the broader impact of treatment. Increasing emphasis on patient-reported outcomes (PROs) has highlighted the need to incorporate patient perspectives into evaluation and quality improvement.

General pediatric quality-of-life and psychological instruments provide useful information, but hypospadias-specific patient-reported outcome measurement remains an active area of development, including conceptual frameworks and prototype instruments such as the Patient Assessment Tool for Hypospadias (PATH) [6,45]. Core-outcome work has also shown substantial heterogeneity in the outcomes reported across studies, reinforcing the need for age-appropriate standardised endpoints that include urinary, sexual, psychosocial, and reproductive domains [46].

Validated patient-reported outcome measures may improve understanding of the relationship between anatomical outcomes and lived experiences. They may also help identify patients who require additional support and provide more meaningful endpoints for future clinical studies and quality improvement initiatives.

Incorporating psychosocial assessment and patient-reported outcomes into routine care represents an important step toward truly patient-centred hypospadias management. A lifelong care model that integrates surgical outcomes with functional, psychological, and quality-of-life measures will provide a more comprehensive understanding of treatment success and better reflect the priorities of patients and families.

## 9. Integrating Emerging Domains into a Future Hypospadias Care Pathway

The selected domains discussed in this review should not be interpreted as mandatory additions to every hypospadias pathway. Rather, they represent complementary options that can be deployed according to phenotype, clinical complexity, patient priorities, and available evidence.

### 9.1. A Proposed Multidisciplinary Care Pathway

**Figure 2:**
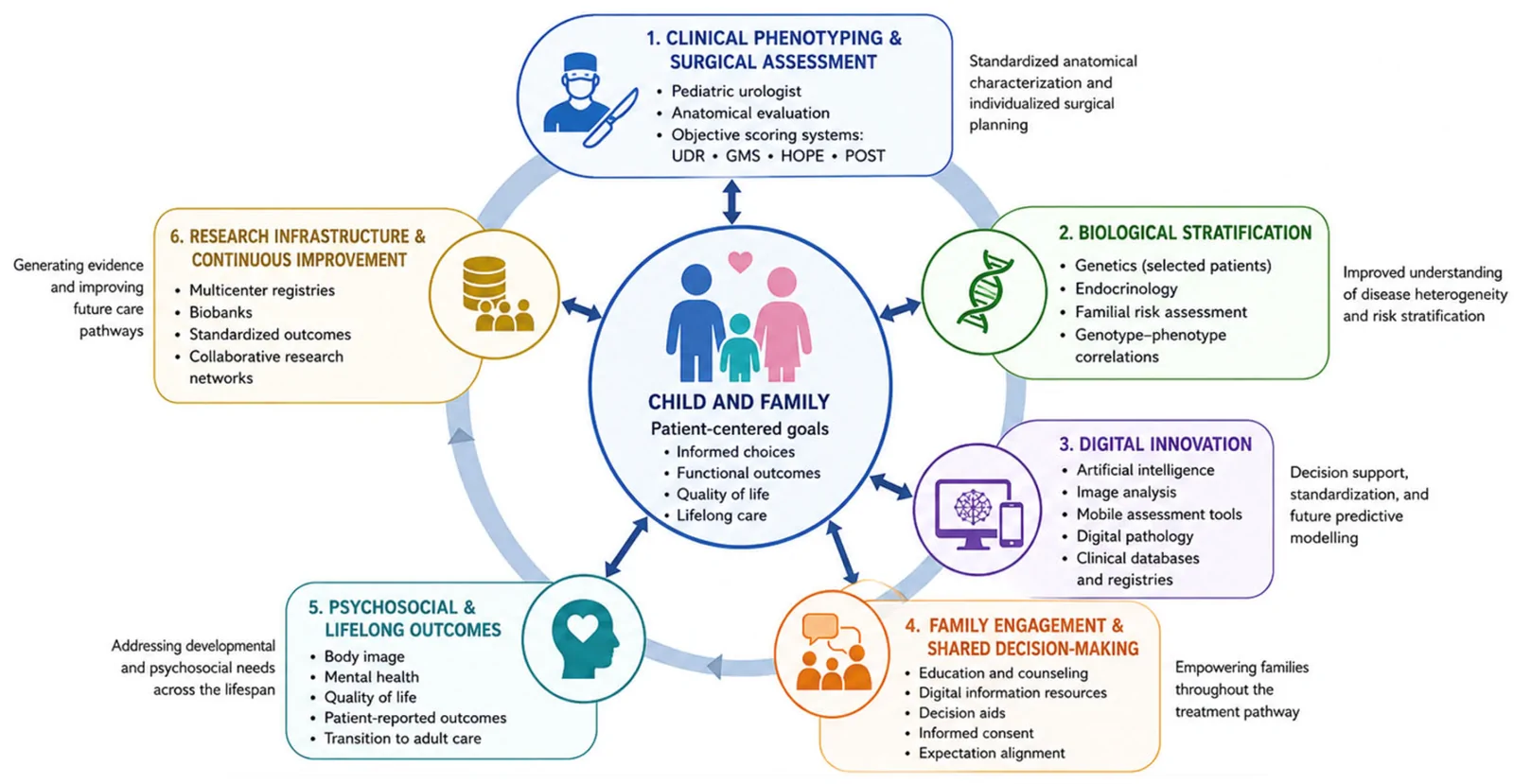
Proposed selective care pathway for contemporary hypospadias management. Routine pediatric urologic care is appropriate for many straightforward distal cases, while proximal/atypical presentations with features suggestive of DSD or other associated conditions may trigger targeted genetic/endocrine and multidisciplinary evaluation. The pathway also incorporates adjunctive digital tools, proportionate family education, longitudinal psychosocial and patient-reported outcome assessment, transition to adult care, and research infrastructure. Digital tools are intended to support—not replace—expert clinical assessment.

The proposed framework places the child and family at the centre of decision-making while distinguishing routine care from selected referral pathways (Figure 2). The model is intended primarily for complex, proximal, atypical, recurrent, or DSD-associated presentations and for longitudinal outcome assessment.

At initial assessment, standardised anatomical phenotyping provides objective characterisation of disease complexity. Families of boys with proximal hypospadias should be offered a discussion of endocrine/genetic evaluation, which is most strongly indicated when associated findings are present; when DSD is suspected, multidisciplinary assessment should follow. If DSD is excluded and no other indication exists, ongoing management can remain within pediatric urology. Digital technologies—including AI-assisted assessment, digital pathology, and clinical databases—remain adjunctive research or decision-support tools rather than requirements for routine care. Proposed service components are summarised in Table 2.

Family education should be proportionate to the complexity and preference-sensitivity of the case. In complex or recurrent disease, structured decision support and psychosocial input may be valuable. Longitudinal follow-up should address urinary function, sexual health, fertility, body image, and patient-reported outcomes, with transition to adult care when clinically appropriate. Registries and biobanks can support research into genotype–phenotype relationships and long-term outcomes.

### 9.2. Challenges to Implementation

Despite the conceptual advantages of multidisciplinary care, implementation requires careful adaptation to local healthcare environments. Barriers include:

- Availability of specialised personnel;
- Differences in healthcare infrastructure;
- Financial constraints;
- Unequal access to advanced diagnostics and digital technologies;
- Challenges in maintaining long-term follow-up.

Future models should therefore prioritise scalable approaches, including regional referral networks, telemedicine-supported collaboration, standardised assessment tools, shared educational resources, and clear criteria for escalation to genetics/endocrinology/DSD services.

### 9.3. Research Priorities for the Next Generation of Hypospadias Care

The proposed framework highlights several priorities requiring further investigation:

1. Prospective multicentre registries integrating anatomical, genetic, surgical, and patient-reported outcomes;
2. Standardised phenotyping approaches to improve disease classification and facilitate comparison between centres;
3. Prospective validation of AI-based tools across diverse populations before clinical adoption;
4. Longitudinal studies evaluating adult outcomes, including sexual health, quality of life, and psychological well-being;
5. Health economic evaluation of multidisciplinary clinics to determine feasibility, sustainability, and value;
6. Prospective studies comparing operative and non-operative management in carefully phenotyped mild distal hypospadias, including patient-reported and sexual outcomes;
7. Studies integrating GWAS, single-nucleotide polymorphism (SNP)-based susceptibility, whole-exome/other sequencing, embryological phenotyping, and environmental exposures;
8. Longitudinal transition pathways and adult reconstructive services addressing late complications, fertility, paternity, sexual health, and patient priorities.

Future advances will depend not on a single innovation, but on evidence-based selection of complementary approaches, careful distinction between routine distal care and complex/DSD-associated disease, and sufficiently long follow-up to capture outcomes that matter to patients.

## 10. Conclusions

This selected narrative review supports a phenotype-driven approach to contemporary hypospadias care. Objective phenotyping may complement meatal classification; endocrine and genetic evaluation should be considered for boys with proximal hypospadias, including those with descended testes, and is most strongly indicated when atypical features suggest DSD or another associated disorder; and straightforward distal hypospadias should not automatically be placed within a multidisciplinary pathway. Limited evidence also supports explicit discussion of non-operative management in selected mild distal cases, alongside greater attention to the uncertainty surrounding long-term outcomes.

The review further highlights the importance of lifelong outcome assessment, including urinary function, sexual health, fertility and paternity, psychosocial well-being, and late surgical complications. AI, digital pathology, registries, and biobanks remain promising but investigational. Future research should prioritise prospective multicentre validation, standardised outcomes, transparent selection criteria for multidisciplinary evaluation, and longitudinal studies extending into adulthood.

## Figures and Tables

**Table 1:** Emerging domains in contemporary hypospadias care.

Domain	Current Application	Evidence Status	Future Direction
Objective phenotyping and severity assessment	Standardised evaluation of anatomical complexity using parameters beyond meatal location, including urethral plate characteristics, curvature assessment, and scoring systems such as UDR, GMS, HOPE, and POST	Increasing evidence supporting improved characterisation of anatomical heterogeneity and interobserver consistency; further validation across centres is ongoing	Development of integrated phenotyping models combining anatomical, biological, and clinical variables for individualised risk stratification
Genetic and biological stratification	Endocrine/genetic evaluation considered in proximal hypospadias, including with descended testes, and prioritised when accompanied by cryptorchidism, micropenis, undervirilisation, familial disease, or features suggestive of DSD; genomic research includes GWAS and sequencing approaches	Growing evidence for genetic heterogeneity and susceptibility loci; clinical diagnostic yield remains variable and interpretation can be challenging	Integrate genotype, embryology, phenotype, endocrine findings, and longitudinal outcomes; define clearer criteria for clinical testing
Prenatal diagnosis and imaging	Prenatal identification using ultrasonography; selected use of fetal MRI in complex or ambiguous genital presentations	Ultrasound remains the primary diagnostic modality; advanced imaging provides additional anatomical information in selected cases	Improved prenatal risk assessment, standardised imaging protocols, and coordinated prenatal counselling pathways
Artificial intelligence and digital technologies	AI-assisted image classification, automated assessment of hypospadias parameters, mobile-based assessment tools, clinical decision support systems, and digital platforms such as AI-PEDURO	Early validation studies demonstrate potential for improving consistency and accessibility; clinical implementation remains investigational	Development and multicentre validation of predictive models, integration into clinical workflows, and responsible deployment in diverse healthcare settings
Digital histopathology and tissue assessment	Quantitative analysis of urethral plate tissue characteristics, including fibrosis, inflammation, and other microscopic features using digital image-based approaches	Emerging evidence suggests potential value as an objective assessment tool; clinical utility requires further validation	Identification of tissue biomarkers that may assist surgical decision-making and prediction of healing outcomes
Family education and shared decision-making	Structured counselling, educational resources, digital platforms, decision aids, and communication strategies to improve understanding of diagnosis and treatment options	Evidence supports the importance of informed communication in reducing uncertainty and improving family engagement; optimal implementation models vary	Development of standardised, culturally adapted educational resources and validated decision-support interventions
Psychosocial care and patient-reported outcomes	Assessment of body image, psychological well-being, quality of life, sexual health concerns, and transition needs during adolescence and adulthood	Increasing recognition of psychosocial outcomes as important endpoints; hypospadias-specific validated tools remain limited	Development of condition-specific patient-reported outcome measures and integration of longitudinal psychosocial assessment into routine care
Multi disciplinary care models	Targeted collaboration for DSD-suspected, complex, atypical, recurrent, or otherwise clinically indicated cases; routine distal cases generally remain within pediatric urology	Conceptually useful in selected complex cases; comparative effectiveness and optimal referral thresholds remain incompletely established	Define phenotype-based referral criteria, outcomes, cost-effectiveness, and scalable regional models
Research infrastructure and learning health systems	Prospective registries, biobanks, standardised databases, and multicentre collaborative research networks	Early development with increasing recognition of the importance of longitudinal data collection	Integration of clinical, biological, technological, and patient-reported data to enable continuous improvement and discovery

AI, artificial intelligence; AI-PEDURO, Artificial Intelligence in Pediatric Urology; DSD, disorders of sex development; GMS, Glans–Urethral Meatus–Shaft score; GWAS, genome-wide association study; HOPE, Hypospadias Objective Penile Evaluation; MRI, magnetic resonance imaging; POST, Plate Objective Scoring Tool; UDR, urethral defect ratio.

**Table 2:** Proposed components of a selective multidisciplinary service for complex, atypical, recurrent, or DSD-associated hypospadias.

Specialty/Component	Role Within Multidisciplinary Hypospadias Care
Pediatric urology	Central coordination of diagnosis, anatomical assessment, surgical planning, operative management, complication management, and longitudinal follow-up. Integration of standardised phenotyping systems (UDR, GMS, HOPE, POST) to support individualised care pathways.
Pediatric and reconstructive surgery	Collaboration in complex reconstructive cases, staged procedures, and management of challenging anatomy or redo repairs when required.
Clinical genetics	Evaluation of proximal/atypical or familial cases, prioritising patients with cryptorchidism, micropenis, undervirilisation, or features suggestive of DSD; interpretation of results and counselling.
Pediatric endocrinology	Assessment of suspected hormonal disorders or DSD when clinically indicated; not required routinely for isolated distal hypospadias.
Radiology/prenatal medicine	Support for prenatal diagnosis, selected advanced imaging in complex cases, and coordination of prenatal counselling and postnatal referral pathways.
Psychology/mental health services	Support when psychosocial concerns, body-image issues, decisional conflict, anxiety, or transition needs are identified; intensity should be individualised.
Specialised nursing/clinical coordinators	Facilitation of patient navigation, education, appointment coordination, postoperative care instructions, communication between specialties, and continuity throughout the care pathway.
Patient education and family support services	Development of educational materials and decision aids for complex or preference-sensitive cases; routine counselling remains appropriate for straightforward distal disease.
Pathology/digital pathology expertise	Evaluation of tissue characteristics when available, including urethral plate biology, fibrosis, and inflammatory changes; collaboration in developing potential tissue-based biomarkers.
Artificial intelligence and data science teams	Development and validation of AI-assisted assessment tools, digital decision-support systems, image analysis platforms, and predictive models while ensuring responsible implementation.
Research and biobank infrastructure	Establishment of prospective registries, biobanks, standardised databases, and multicentre collaborations to support translational research and continuous improvement.
Adult urology/transition care services	Support transition from pediatric to adult healthcare, addressing long-term urinary function, sexual health, reproductive concerns, and patient-reported outcomes.
Administrative and institutional support	Ensuring sustainable multidisciplinary clinic organisation, resource allocation, quality monitoring, and integration within healthcare systems.

GMS, Glans–Urethral Meatus–Shaft score; HOPE, Hypospadias Objective Penile Evaluation; POST, Plate Objective Scoring Tool; UDR, urethral defect ratio.

## Data Availability

No new data were created or analysed in this study. Data sharing is not applicable to this article.

## Abbreviations

The following abbreviations are used in this manuscript:

AI	Artificial intelligence
AI-PEDURO	Artificial intelligence in pediatric urology
AR	Androgen receptor
DSD	Disorders of sex development
GMS	Glans–urethral meatus–shaft score
GWAS	Genome-wide association study
HOPE	Hypospadias Objective Penile Evaluation
MRI	Magnetic resonance imaging
PATH	Patient Assessment Tool for Hypospadias
POST	Plate Objective Scoring Tool
PRO	Patient-reported outcome
SNP	Single-nucleotide polymorphism
SRY	Sex-determining region Y
UDR	Urethral defect ratio
